# Fat-Soluble Vitamin Deficiency in Pediatric Patients with Biliary Atresia

**DOI:** 10.1155/2017/7496860

**Published:** 2017-06-11

**Authors:** Rui Dong, Song Sun, Xiao-Zhou Liu, Zhen Shen, Gong Chen, Shan Zheng

**Affiliations:** Department of Pediatric Hepatobiliary Surgery, Children's Hospital of Fudan University and Key Laboratory of Neonatal Disease, Ministry of Health, 399 Wan Yuan Road, Shanghai 201102, China

## Abstract

**Objective:**

To analyze the levels of fat-soluble vitamins (FSVs) in pediatric patients with biliary atresia (BA) before and after the Kasai procedure.

**Methods:**

Pediatric patients with obstructive jaundice were enrolled in this study. The FSV levels and liver function before, 2 weeks after, and 1, 3, and 6 months after the Kasai procedure were measured.

**Results:**

FSV deficiency was more obvious in patients with BA than in patients with other cholestatic liver diseases, especially vitamin D deficiency. 25-Hydroxy vitamin D (25-(OH)D) deficiency was more pronounced in younger patients before surgery. The 25-(OH)D level was significantly higher in patients with than without resolution of jaundice 3 months after surgery. At 6 months after surgery, the 25-(OH)D level was abnormally high at 8.76 ng/ml in patients with unresolved jaundice.

**Conclusions:**

Preoperative FSV deficiency, particularly vitamin D deficiency, is common in patients with BA. 25-(OH)D deficiency is more pronounced in younger children before surgery. Postoperative FSV deficiency was still prevalent as shown by the lower 25-(OH)D levels in patients with BA and unresolved jaundice. This required long-term vitamin AD supplementation for pediatric patients with BA and unresolved jaundice after surgery.

## 1. Introduction

Biliary atresia (BA) is defined as biliary obstruction caused by progressive fibrosis of intrahepatic and extrahepatic bile ducts with an unknown pathogenesis. If not treated promptly, it will inevitably lead to liver cirrhosis and liver failure, and affected patients often die within 12 to 18 months after birth [1, 2]. The liver plays a central role in regulating the balance of nutrients, and liver diseases lead to abnormal nutrient metabolism in the body and thereby to nutritional disorders [3, 4]. Even if the Kasai procedure is successfully performed, many pediatric patients with BA may still have an abnormal nutritional status and developmental delays that result from irreversible BA-induced liver damage. Thus, the mortality rate increases in the later stages, along with earlier liver transplantation. Regular nutrition assessments are therefore necessary for children with BA [5]. The uptake, distribution, storage, and use of fat-soluble vitamins (FSVs) are closely associated with liver function [6, 7]. FSV abnormalities are particularly prominent in pediatric patients with BA with malnutrition because of damage to liver function, and FSV deficiency can reflect changes in liver function [8].

Vitamins are a group of substances that are essential for maintaining the normal physiological function of the body. FSVs are closely involved in the processes of antioxidation, blood coagulation, and calcium/phosphorus uptake in the human body. FSVs include vitamins A, D, E, and K, which are mainly stored and metabolized in the liver. After absorption into the intestinal cells, the vitamins are wrapped in chylomicrons and enter the lymph system before they are transported into the liver, where they are metabolized. Some products of their metabolism are stored in the liver, while others enter the blood circulation and are transported to target tissues or cells. Bile acid is essential for the normal uptake of FSVs. Pediatric patients with BA have abnormal biliary excretion, liver function damage, and abnormal FSV uptake and metabolism. Even after a successful surgery, the hepatic secretion of bile acid remains impaired for a long time [9]. Bile ducts in these patients are almost completely blocked several months before surgery, and even after a successful Kasai procedure, bile acid secretion in the liver does not reach normal levels within 6 to 12 months. Because of progressive liver function damage in patients with BA, the liver's ability to convert and store vitamins and produce serum albumin is inevitably affected. Deficiencies in vitamin A-binding protein, vitamin D-binding protein, and several lipoproteins will lead to a decrease in vitamin vectors in the bloodstream, which can also cause disordered systemic use of FSV [10]. In addition, the amount of bilirubin in the blood can affect FSV uptake [11, 12].

In the current study, we assessed the FSV levels and liver function in patients with BA to determine the relationship of FSV deficiency before and after surgery with liver function. These findings will guide clinical nutrient supplementation and nutrition monitoring in patients with BA.

## 2. Experimental Procedures

### 2.1. Patients and Inclusion Criteria

Pediatric patients who were admitted because of obstructive jaundice (including patients in whom BA was confirmed during surgery and patients with cholestasis but without BA) from January 2014 to December 2014 were enrolled in this study.

The inclusion criteria for pediatric patients with BA were confirmation of BA by intraoperative cholangiography and no other severe systematic deformity (such as BA splenic malformation syndrome). The exclusion criteria were bile duct dysplasia and/or malformation of other systems.

The inclusion criteria for pediatric patients with cholestasis were cholestasis without BA as confirmed by intraoperative cholangiography and no other severe malformation in other systems.

### 2.2. Research Methods

Demographic information (including sex, weight, and age at the time of surgery) as well as the FSV levels and liver function before surgery (*n* = 221), 2 weeks after surgery (*n* = 221), and 1 (*n* = 218), 3 (*n* = 210), and 6 (*n* = 201) months after surgery was recorded. Percentages of FSV deficiencies in patients with BA before surgery were calculated and compared with those in patients with cholestasis during the same period. The changes in the FSV levels before and after surgery in patients with BA were also analyzed and compared with the change in liver function. In patients who underwent the Kasai procedure, one vitamin AD gel capsule (each containing 2000 IU of vitamin A and 3700 IU of vitamin D) was administered daily beginning on postoperative day 4 (resumption of oral diet) to 1 month after surgery.

The patients with BA were divided by sex, and the relationship between FSV deficiency and sex was analyzed. The patients with BA were also divided into three age groups: 30 to 60, 60 to 90, and >90 days of age. The preoperative FSV deficiency rate in each group was calculated, and the difference in FSV deficiency among these age groups was compared to determine the potential impact of the disease course on FSV deficiency. In the BA group, the potential correlation between the preoperative FSV level and liver function-related indicators was analyzed to determine the possible correlations between the change in FSV and the change in liver function.

Patients were grouped as follows according to postoperative jaundice resolution. (1) One month after the surgery, the patients were divided according to their total bilirubin (TB) levels into a low-bilirubin group (TB ≤ 51.3 *μ*mol/L) and a high-bilirubin group (TB> 51.3 *μ*mol/L), and the changes in FSVs (including vitamins A, D, and E and 25-hydroxy vitamin D (25-(OH)D)) were compared between these two groups. (2) Three months after the surgery, the patients were divided into a jaundice-resolved group (direct bilirubin ≤ 17.1 *μ*mol/L) and a jaundice-unresolved group (direct bilirubin > 17.1 *μ*mol/L), and the changes in FSVs (including vitamins A, D, and E and 25-(OH)D) were compared between these two groups. (3) Six months after the surgery, the changes in FSVs (including vitamins A, D, and E and 25-(OH)D) were compared between the jaundice-resolved group (direct bilirubin ≤ 17.1 *μ*mol/L) and jaundice-unresolved group (direct bilirubin > 17.1 *μ*mol/L).

### 2.3. Statistical Methods

The statistical methods were selected based on the samples. The Shapiro-Wilk normality test was performed for all continuous variables before the analysis. All variables were nonnormally distributed, so medians and interquartile ranges were used to describe the distribution of variables. The Wilcoxon rank sum test was used to compare continuous variables between the two groups. Comparisons among multiple groups were based on the Kruskal-Wallis test. Discrete variables are presented using frequencies and percentages. The chi-square test was applied for intergroup comparisons, and Pearson's chi-square or Fisher's exact test was used based on the expected cell counts. Correlations between variables were analyzed using Spearman's correlation coefficient. A value of *P* < 0.05 was considered statistically significant. All statistical analyses were performed using SAS 9.3 software (SAS Institute, Cary, NC, USA).

## 3. Results

### 3.1. Preoperative FSV Deficiencies in Patients with BA

#### 3.1.1. Age and Sex of Patients with BA and Patients with Cholestasis

In total, 266 pediatric patients with obstructive jaundice were enrolled in this study. Among these patients, 221 had BA and 45 had cholestasis. There was no difference in age between these two groups (*P* > 0.48), and there were significantly more males than females (Table 1).

#### 3.1.2. FSV Deficiencies in Patients with BA and Patients with Cholestasis

The overall FSV deficiencies in pediatric patients with obstructive jaundice are shown in Supplementary Table 1 available online at https://doi.org/10.1155/2017/7496860. The highest rate of 25-(OH)D deficiency was 87.8%. In the BA group, the rate of 25-(OH)D deficiency was 88.3%, and the rate of one or more vitamin deficiencies was 45.9% (Supplementary Table 2). In the cholestasis group, the rate of 25-(OH)D deficiency was 85.7%, and the rate of one or more vitamin deficiencies was 20.0% (Supplementary Table 3).

Compared with the cholestasis group, the BA group had more severe overall FSV deficiencies. This manifested as a significant difference in the rate of one or more vitamin deficiencies (45.9% versus 20.0%, *P* = 0.0014), suggesting that the proportion of patients with several vitamin deficiencies was higher in the BA group. Further analysis showed that the rate of vitamin D deficiency was 31.3% in the BA group and 6.7% in the cholestasis group (*P* = 0.0007). The proportion of patients with a prolonged prothrombin time (PT) was significantly lower in the BA than in the cholestasis group (6.0% versus 15.6%, *P* = 0.061), whereas deficiencies in vitamin A (15.6% versus 13.3%, *P* = 0.69), 25-(OH)D (88.3% versus 85.7%, *P* = 0.76), and vitamin E (4.3% versus 2.2%, *P* = 0.81) were not significantly different between these two groups (Supplementary Table 4).

### 3.2. Factors Related to Preoperative FSV Deficiency in Patients with BA

#### 3.2.1. Relationship between Preoperative FSV Deficiency and Sex

The 221 pediatric patients with BA comprised 115 males and 106 females. There was no significant difference in any variables between males and females, suggesting that the preoperative FSV deficiency was not significantly correlated with sex in children with BA (Supplementary Table 5).

#### 3.2.2. Relationship between Preoperative FSV Deficiency and Age

Vitamin A deficiency was significantly different among the three age groups (*P* = 0.05), and the rate of vitamin A deficiency decreased as age increased. Similarly, 25-(OH)D deficiency also significantly differed among the different age groups (*P* = 0.0096), and this deficiency rate remarkably decreased as age increased (Supplementary Table 6).

#### 3.2.3. Comparison of Liver Function between BA and Cholestasis Groups

Comparison of liver function between the BA and cholestasis groups (Supplementary Tables 7–9) showed that while both groups had obstructive jaundice, the levels of TB, direct bilirubin, and bile acids were significantly higher in the BA than in the cholestasis group (*P* < 0.022, *P* = 0.035, and *P* = 0.058, resp.). The BA group also had a significantly higher *γ*-glutamyl transferase level than the cholestasis group (*P* < 0.0001). Liver function impairment was present in both groups, but it was more severe in the BA than in the cholestasis group. The BA group had a significantly higher increase in the aspartate transaminase (AST) level than did the cholestasis group (*P* < 0.040). Because FSV deficiency was also more severe in the BA than in the cholestasis group, FSV deficiency might be correlated with the severity of cholestasis and liver function impairment.

#### 3.2.4. Comparison of Liver Function among Different Age Groups in Patients with BA

The direct bilirubin level was not significantly different among the three age groups of patients with BA. However, the alkaline phosphatase (ALP), *γ*-glutamyl transferase, alanine aminotransferase (ALT), AST, and bile acid levels were significantly different among these age groups and increased as age increased. Liver function progressively worsened if bile duct obstruction persisted. An older age at surgery was associated with a greater possibility of liver function impairment; however, the rate of FSV deficiency decreased with increasing age, which might be explained by the patients' increased awareness of dietary supplementation and/or the body's own compensatory mechanisms (Supplementary Table 10).

### 3.3. Relationship between Preoperative FSV Deficiency and Liver Function in Patients with BA

#### 3.3.1. Relationship between Abnormal Preoperative 25-(OH)D Level and Liver Function in Patients with BA

The 25-(OH)D level was positively correlated with *γ*-glutamyl transferase and bile acid (*r* = 0.223, *P* = 0.031; *r* = 0.210, *P* = 0.042), suggesting that the metabolism and uptake of 25-(OH)D is closely related to the change in the *γ*-glutamyl transferase level in that an increase in *γ*-glutamyl transferase significantly affects 25-(OH)D metabolism and uptake. In addition, the 25-(OH)D level was positively correlated with serum calcium. Serum calcium levels might decrease as the vitamin D level decreases, but the serum calcium level was 2.5 mmol/L (range, 2.4–2.6 mmol/L; normal range, 2.25–2.75 mmol/L), suggesting that the serum calcium level was within the normal range in most patients. The 25-(OH)D level did not show theoretically negative correlations with liver function. This may be because our patients had obviously abnormal liver function and high bilirubin, ALP, and *γ*-glutamyl transferase levels. However, there was no linear correlation between 25-(OH)D and liver function (Supplementary Table 11).

#### 3.3.2. Relationship between Prolonged Preoperative PT and Liver Function in Patients with BA

The PT was positively correlated with the changes in ALP, ALT, and AST (*r* = 0.310, *P* < 0.0001; *r* = 0.208, *P* = 0.002; *r* = 0.232, *P* = 0.001) and negatively correlated with *γ*-glutamyl transferase and albumin (*r* = −0.291, *P* < 0.0001; *r* = −0.381, *P* < 0.0001). However, the PT showed no significant correlation with bilirubin (Supplementary Table 12).

### 3.4. Changes in FSV Levels after the Kasai Procedure in Patients with BA

#### 3.4.1. Changes in Serum FSV before and after the Kasai Procedure in Patients with BA

**Figure 1:**
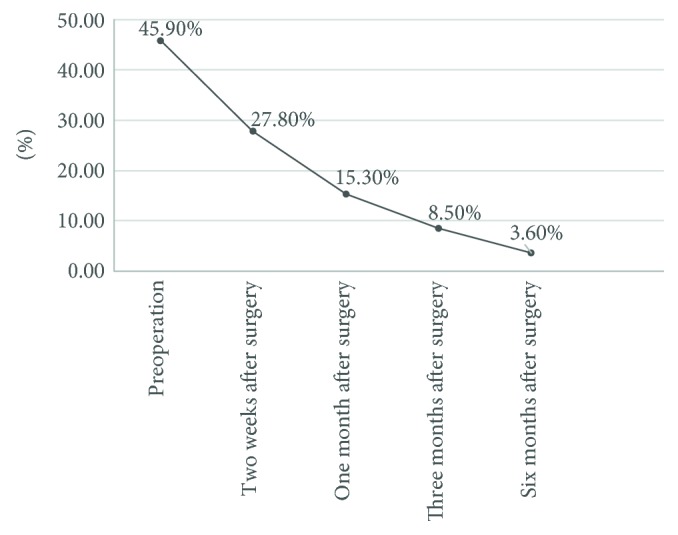
Changes in serum FSVs before and after the Kasai procedure in patients with BA. The proportions of patients with a deficiency in vitamins A, D, and E or one or more vitamins were 27.8%, 15.3%, 8.5%, and 3.6% at 2 weeks and 1, 3, and 6 months after surgery, respectively, showing significant improvement in the percentage of patients with one or more vitamin deficiencies (preoperative value, 45.9%; *P* < 0.0001).

Some patients still had FSV deficiency after the Kasai procedure. The proportions of patients with a deficiency of vitamins A, D, and E or one or more vitamins were 27.8%, 15.3%, 8.5%, and 3.6% at 2 weeks and 1, 3, and 6 months after surgery, respectively, showing significant improvement in the percentage of patients with one or more vitamin deficiencies (preoperative value, 45.9%; *P* < 0.0001) (Figure 1).

#### 3.4.2. Changes in Serum Vitamin A Level before and after the Kasai Procedure in Patients with BA

The percentage of patients with vitamin A deficiency was 16.3% before surgery and changed to 16.7%, 8.5%, 3.4%, and 6.8% at 2 weeks and 1, 3, and 6 months after surgery, respectively. It remained high 2 weeks after surgery, suggesting that the bile flow had not been established in most patients and that surgery-induced damage to liver function was still present. The mean postoperative vitamin A level was within the normal range (0.52–2.2 *μ*mol/L) (Supplementary Table 13).

#### 3.4.3. Changes in Serum Vitamin D Level before and after the Kasai Procedure in Patients with BA

The percentage of vitamin D deficiency was 15.3% before surgery and 5.6%, 5.1%, 6.8%, and 3.6% at 2 weeks and 1, 3, and 6 months after surgery, respectively. Thus, the percentage of vitamin D deficiency was significantly improved after surgery. The mean serum vitamin D level was not significantly changed in patients with BA (normal range, 25–200 nmol/L) (Supplementary Table 14).

#### 3.4.4. Changes in Serum 25-(OH)D Level before and after the Kasai Procedure in Patients with BA

The percentage of 25-(OH)D deficiency was 88.3% before surgery and 93.8%, 91.5%, 49.1%, and 53.6% at 2 weeks and 1, 3, and 6 months after surgery, respectively. Thus, the 25-(OH)D deficiency was remarkably improved 3 and 6 months after surgery. The mean value of 25-(OH)D was below the normal range (15–35 ng/ml) but reached the lower threshold of the normal range 3 months after surgery. It dropped to the lowest level 2 weeks after surgery and remarkably increased 3 and 6 months after surgery, suggesting that the recovery time was prolonged after the Kasai procedure and that the mean serum 25-(OH)D level showed an increasing trend (Supplementary Table 15).

#### 3.4.5. Changes in Serum Vitamin E Level before and after the Kasai Procedure in Patients with BA

The percentage of vitamin D deficiency was 4.1% before surgery and 8.3%, 1.7%, 1.7%, and 0.0% at 2 weeks and 1, 3, and 6 months after surgery, respectively. Thus, vitamin E deficiency was remarkably improved 3 and 6 months after surgery. The change in serum vitamin E levels before and after surgery was not obvious, and the level remained within the normal range (10–15 ng/ml) (Supplementary Table 16).

### 3.5. Correlation between FSV Levels and Bilirubin Changes before and after Surgery in Patients with BA

#### 3.5.1. Relationship between FSV Levels and Bilirubin Changes before and after Surgery in Patients with BA

**Figure 2:**
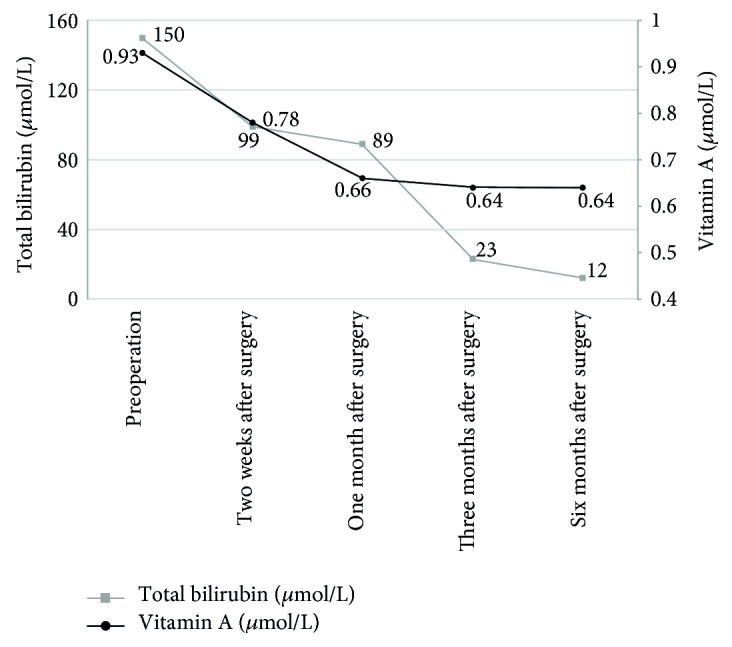
Relationship between FSV levels and bilirubin changes before and after surgery in patients with BA. The median serum vitamin A level did not increase postoperatively. It was 0.93, 0.78, 0.66, 0.64, and 0.64 *μ*mol/L before surgery, 2 weeks after surgery, and 1, 3, and 6 months after surgery, respectively; these values were within the normal range (0.52–2.20 *μ*mol/L). The TB level decreased gradually postoperatively. It was 150.5, 99.25, 89, 22.6, and 12 *μ*mol/L before surgery, 2 weeks after surgery, and 1, 3, and 6 months after surgery, respectively, showing no significant difference between the two groups. Thus, although the bile flow was established after surgery and the TB level remarkably decreased, the vitamin A level did not change significantly within the short follow-up time after surgery.

The median serum vitamin A level did not increase postoperatively. It was 0.93, 0.78, 0.66, 0.64, and 0.64 *μ*mol/L before surgery, 2 weeks after surgery, and 1, 3, and 6 months after surgery, respectively; all of these levels were within the normal range (0.52–2.20 *μ*mol/L). The TB decreased gradually postoperatively. It was 150.50, 99.25, 89.00, 22.60, and 12.00 *μ*mol/L before surgery, 2 weeks after surgery, and 1, 3, and 6 months after surgery, respectively, showing no significant difference between the two groups. Thus, although the bile flow was established after surgery and the TB level remarkably decreased, the vitamin A level did not change significantly within the short follow-up time after surgery (Figure 2).

#### 3.5.2. Relationship between 25-(OH)D Level and Bilirubin Change before and after Surgery in Patients with BA

**Figure 3:**
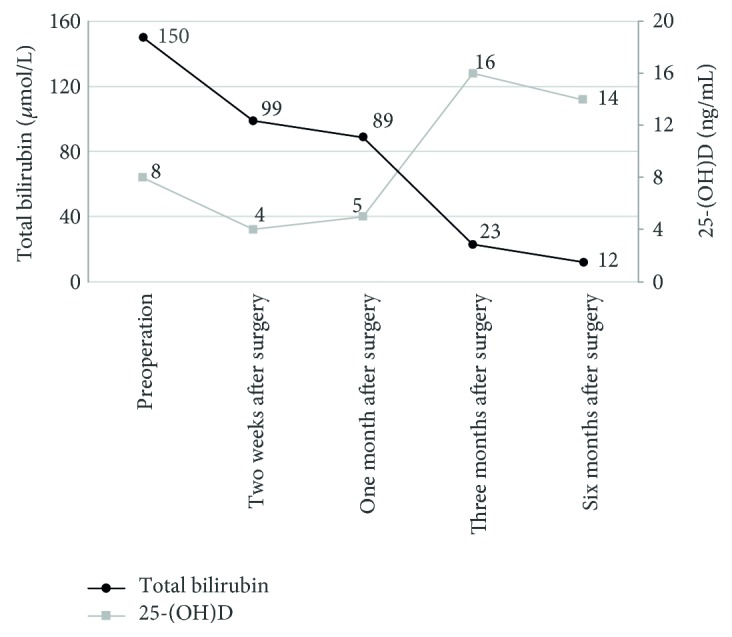
Relationship between 25-(OH)D level and bilirubin change before and after surgery in patients with BA. The median serum 25-(OH)D level was 7.66, 3.04, 5.13, 15.55, and 13.91 ng/ml before surgery, 2 weeks after surgery, and 1, 3, and 6 months after surgery, respectively. After adjustment for the time variable, the 25-(OH)D level was negatively correlated with the TB level, suggesting that the uptake and metabolism of 25-(OH)D were remarkably improved after the serum TB level decreased after surgery.

The median serum 25-(OH)D level was 7.66, 3.04, 5.13, 15.55, and 13.91 ng/ml before surgery, 2 weeks after surgery, and 1, 3, and 6 months after surgery, respectively. After adjusting for the time variable, the 25-(OH)D level was negatively correlated with the TB level (*P* < 0.0001), suggesting that the uptake and metabolism of 25-(OH)D were remarkably improved after the serum TB decreased after surgery (Figure 3).

### 3.6. Differences in FSV Levels between Groups with Different Changes in TB after Surgery

#### 3.6.1. Differences in FSV Levels between the Low- and High-Bilirubin Group 1 Month after Surgery

One month after the Kasai procedure, there were 73 patients (33.5%) in the low-bilirubin group (TB < 51.3 *μ*mol/L) and 145 patients (66.5%) in the high-bilirubin group (TB > 51.3 *μ*mol/L). Although the vitamin A level was significantly lower in the low-bilirubin group (*P* = 0.0008), it was within the normal range in both groups. In addition, vitamin E, vitamin D, and 25-(OH)D levels were not significantly different between the low- and high-bilirubin groups 1 month after surgery. Thus, the changes in TB had little impact on FSV levels 1 month after surgery (Supplementary Table 17).

#### 3.6.2. Comparison of FSV Levels between Jaundice-Resolved and Jaundice-Unresolved Groups 3 Months after Surgery

Three months after the Kasai procedure, there were 120 patients (120/210, 57.1%) in the jaundice-resolved group (direct bilirubin ≤ 17.1 *μ*mol/L) and 90 patients (90/210, 42.9%) in the jaundice-unresolved group (direct bilirubin > 17.1 *μ*mol/L). The jaundice-resolved group had a significantly lower serum vitamin A level than the jaundice-unresolved group (*P* = 0.0007). There was no significant difference in the vitamin E or D level between these two groups (*P* = 0.57). However, the 25-(OH)D level was significantly higher in the jaundice-resolved than in the jaundice-unresolved group (*P* = 0.0016), suggesting that jaundice resolution 3 months after surgery can improve the 25-(OH)D level (Supplementary Table 18).

#### 3.6.3. Comparison of FSV Levels between the Jaundice-Resolved and Jaundice-Unresolved Groups 6 Months after Surgery

Six months after the Kasai procedure, there were 139 patients (139/201, 69.2%) in the jaundice-resolved group and 62 patients (62/201, 30.8%) in the jaundice-unresolved group. The vitamin A level was significantly lower in the jaundice-resolved than in the jaundice-unresolved group (*P* = 0.017). The vitamin E level was not significantly different between these two groups (*P* = 0.93). The vitamin D level was significantly higher in the jaundice-resolved than in the jaundice-unresolved group (*P* = 0.0012). The jaundice-resolved group had a significantly higher 25-(OH)D level than in the jaundice-unresolved group (*P* = 0.0006), suggesting that recovery of the 25-(OH)D level was better in the jaundice-resolved than in the jaundice-unresolved group. The mean 25-(OH)D level in the jaundice-unresolved group remained below the normal range 6 months after surgery (Supplementary Table 19).

## 4. Discussion

Vitamins are essential for maintaining normal physiological function of the body. FSVs are closely involved in antioxidation, blood coagulation, and calcium/phosphorus uptake in the human body. Vitamin deficiency, particularly FSV deficiency, is common in children with chronic liver diseases; this may be explained by the reduced food intake, impaired nutrient uptake, and reduced synthesis of carrier proteins caused by these patients' damaged liver function [13]. Young et al. [4] reported that the incidence of vitamin deficiency could be 20% to 30% in patients with cholestatic liver disease; this phenomenon is also common in children with BA. Andrews et al. [14] evaluated the levels of vitamins A, D, and E in 29 patients with BA who had undergone hepatic-biliary-enteric anastomosis and found that vitamin deficiency persisted despite surgical reconstruction of the bile flow. A study performed in the United States enrolled 92 patients with BA, and detection of FSV, retinol-binding protein, blood lipids, and TB at 1, 3, and 6 months after the Kasai procedure showed that FSV deficiency was common; the percentages of vitamins A, D, K, and E deficiency were 29% to 36%, 21% to 37%, 10% to 22%, and 16% to 18%, respectively [15]. Cywes and Millar [16] found that the serum levels of vitamins A, E, and D were significantly decreased in 11 children with BA. In the current study, FSV deficiency was common among 266 pediatric patients with obstructive jaundice before surgery. The percentage of patients with one or more vitamin deficiencies was 45.9% in the BA group and 20.0% in the cholestasis group, and this difference was statistically significant. Vitamin D deficiency was even more severe in the BA than in the cholestasis group (31.3% versus 6.7%). However, the BA group had a significantly lower percentage of patients with a remarkably prolonged PT than did the cholestasis group, suggesting that cholestasis might lead to more severe damage of liver cell function than BA. Thus, cholestasis may have a greater impact on the coagulation mechanism.

In this study, we analyzed the factors related to preoperative FSV deficiency in patients with BA. We found that FSV deficiency was not significantly correlated with sex. Only 25-(OH)D deficiency was significantly different among the three age groups, and the rate of this deficiency remarkably decreased as age increased. The serum 25-(OH)D concentration is often used to determine the vitamin D status in the human body, and 25-(OH)D has a relatively long half-life (15 days) in the human circulation; however, the blood level of 25-(OH)D does not reflect the vitamin D level in other tissues [17]. The vitamin D level is assessed via the serum 25-(OH)D level because the active form of 1,25-(OH)2-D can be easily stabilized at a normal or high level as a result of renal compensation after small changes in the calcium and phosphorus levels [18, 19]. In addition, the proportion of 25-(OH)D deficiency was decreased 3 and 6 months after the surgery for BA. While the 25-(OH)D level reached the lower threshold of the normal range 3 months after surgery in some patients, it was below the normal range (15–35 ng/ml) in others. It dropped to its lowest level 2 weeks after surgery and then remarkably increased 3 and 6 months after surgery, with the mean serum 25-(OH)D level showing an increasing trend. This suggests that there is a long recovery period after the Kasai procedure. After adjusting for the time variable, analysis of the correlation between the 25-(OH)D and bilirubin levels before surgery, 2 weeks after surgery, and 1, 3, and 6 months after surgery showed that the 25-(OH)D level was negatively correlated with the TB level. This suggests that the 25-(OH)D uptake and metabolism improved along with a decrease in the postoperative serum TB level. Thus, in the younger age group, children with BA were more likely to develop 25-(OH)D deficiency before surgery because of the relatively small reserve of 25-(OH)D. As age increased, the parents would often give appropriate vitamin supplementation to their children; thus, the percentage of 25-(OH)D deficiency decreased. This indicates that preoperative vitamin supplementation is necessary for young infants with an early clinical diagnosis.

Although radical surgery improved biliary drainage in some patients in the present study, liver function was not completely restored and there were still disorders of vitamin uptake and metabolism, which leads to a high incidence of FSV deficiency within a short period of time. All patients were routinely administered one vitamin AD capsule (each contained 2000 IU of vitamin A and 3700 IU of vitamin D) daily beginning on the fourth postoperative day and continuing until 1 month after surgery. FSV deficiency was still present before surgery, 2 weeks after surgery, and 1, 3, and 6 months after surgery. The proportion of patients with vitamin A, D, or E deficiency or a deficiency of one or more vitamins was 27.8%, 15.3%, 8.5%, and 3.6% at 2 weeks and 1, 3, and 6 months after surgery, respectively. The percentage of patients with one or more vitamin deficiencies was significantly improved (preoperative value, 45.9%). Several factors (e.g., establishment of bile flow, dietary supplementation, and increased nutritional education regarding this disease) might contribute to this finding. Early diagnosis of vitamin deficiency can facilitate early nutritional intervention. Because of the lack of simple and effective nutritional screening tools for children, it is difficult to perform basic nutrition screening in pediatric patients [20].

Along with the persistence of cholestasis and the progression of liver damage, patients with BA may have constantly worsening biochemical indicators including increased bilirubin (mainly direct bilirubin), *γ*-glutamyl transferase, ALP, and bile acids with or without an increase in the ALT and/or AST levels. Liver damage was found in all patients with BA in our series. Patients with BA with a TB level of >34 *μ*mol/L had higher risk of FSV deficiency; we found that the vitamin levels were negatively correlated with the serum direct bilirubin level [20]. In the current study, vitamin D deficiency was most prominent in the BA group. Analysis of the potential correlations between the serum 25-(OH)D level and liver function showed that the serum 25-(OH)D level was positively correlated with a change in bile acids; it was also correlated with *γ*-glutamyl transferase. However, our findings were not consistent with some previous studies [19, 21]. Because evidence regarding the correlation between preoperative vitamins and liver function is still lacking, there are not enough data to support specific findings, and additional studies are required to validate the results. The ALP level increases in patients with liver and gallbladder disease, and it is closely associated with bone metabolism [22]. In the present study, 25-(OH)D was positively correlated with serum calcium, indicating that the serum calcium level might decline as vitamin D decreases. During the disease course of a pediatric patient, decreased vitamin D and increased ALP may further affect bone metabolism. Detection of vitamin K deficiency is mainly based on the PT and the international normalized ratio (INR). The PT was abnormal (4.4%) in our study; however, another study also indicated that the PT does not completely reflect the amount of vitamin K and that it might underestimate the percentage of vitamin K deficiency [23]. Analysis of the correlation between the PT and liver function showed that the PT was positively correlated with the changes in ALT and AST, suggesting that vitamin K deficiency worsens along with the deteriorating liver function [24]. Abnormal liver function could also affect the production of other coagulation factors. The PT was negatively correlated with albumin, and the decrease in albumin levels may be associated with a longer PT. The PT was thus correlated with the serum vitamin K level. Albumin is a key carrier protein in the blood [25, 26], and it can also function as a carrier of vitamins. Many children with BA may have an excessively low albumin level early after surgery, which may be explained by preoperative hypoalbuminemia and surgical stress. Therefore, preoperative vitamin K and albumin supplementation to correct coagulation disorders and hypoalbuminemia is valuable for increasing patient tolerance and the surgical success rate and reducing postoperative complications [27].

Although biliary-enteric drainage is established after the Kasai procedure, disorders of the bilirubin excretion may still be present in some patients. In our series, the bilirubin clearance rate was only 33.3% 1 month after surgery, and it reached 56.9% and 69.2% at 3 and 6 months after surgery, respectively. Therefore, hyperbilirubinemia was still present in many patients, and liver function was not completely restored in these patients. Surgical and anesthetic trauma could further damage their liver function, which could affect FSV uptake and metabolism. Kasai surgery is generally an open surgery with an operative time of 1.5 to 2.0 hours and an anesthesia time of about 3.0 hours. The intraoperative bleeding volume varies among patients. Intraoperative blood or albumin transfusion may be performed according to the patient's preoperative protein and hemoglobin levels. The postoperative fasting time is typically 3 to 4 days. Therefore, surgical stress, intraoperative blood loss, and postoperative fasting can affect the nutritional and metabolic status (including FSVs) in pediatric patients. The percentages of vitamin A and 25-(OH)D may remarkably increase 2 weeks after surgery. Even in pediatric patients with normal FSV levels before surgery, the possibility of developing FSV deficiencies is also increased [28]. In children with BA, nutritional abnormalities and lack of energy synthesis and metabolism can also directly or indirectly affect the recovery of liver function and may worsen liver cirrhosis, thereby decreasing the effectiveness of liver transplantation [29].

In conclusion, obvious FSV deficiency is common in pediatric patients with obstructive jaundice. Children with BA have a higher incidence of FSV deficiency (particularly vitamin D deficiency) than children with cholestasis. 25-(OH)D deficiency is more pronounced in younger than in older pediatric patients before surgery. Additionally, 25-(OH)D is positively correlated with serum calcium, indicating that the serum calcium level may decline along with the decrease in vitamin D. In patients with BA, FSV deficiency remains persistent after the Kasai procedure. The 25-(OH)D level remarkably decreases in patients with BA with unresolved jaundice, and long-term postoperative vitamin AD supplementation is required for these patients.

## Supplementary Material

Supplementary Table 1: Overall FSV deficiencies in pediatric patients with obstructive jaundice. Supplementary Table 2: Preoperative FSV deficiencies in BA patients. Supplementary Table 3: Comparisons of FSV deficiencies between BA group and cholestatic group. Supplementary Table 4: Preoperative FSV deficiencies in cholestatic patients. Supplementary Table 5: Relationship between preoperative FSV deficiency and sex in BA patients. Supplementary Table 6: FSV deficiencies in different age groups. Supplementary Table 7: Preoperative liver function in BA patients. Supplementary Table 8: Preoperative liver function in cholestatic patients. Supplementary Table 9: Comparison of liver function between the BA and cholestatic groups. Supplementary Table 10: Comparison of liver function among different age groups in BA patients. Supplementary Table 11: Relationship between abnormal preoperative 25-(OH)D level and liver functions in BA patients. Supplementary Table 12: Relationship between preoperative prothrombin time (PT) and liver function in BA patients. Supplementary Table 13: Changes in serum vitamin A level before and after the Kasai procedure in BA patients. Supplementary Table 14: Changes in the serum vitamin D level before and after the Kasai procedure in BA patients. Supplementary Table 15: Changes of serum 25-(OH)D level before and after the Kasai procedure in BA patients. Supplementary Table 16: Changes in serum vitamin E level before and after the Kasai procedure in BA patients. Supplementary Table 17: FSV levels between the low-bilirubin group and the high-bilirubin group 1 month after surgery. Supplementary Table 18: Comparison of vitamin levels between the jaundice-cleared group and the jaundice-non-resolved group 3 months after surgery. Supplementary Table 19: Comparison of vitamin levels between the jaundice-cleared group and the jaundice-non-resolved group 6 months after surgery.

## Figures and Tables

**Table 1:** Sex of patients with biliary atresia and patients with cholestasis.

Sex	Biliary atresia		Cholestasis		*X*	
	Frequency	Percentage	Frequency	Percentage		*P* value
Male	115	52.0%	33	73.3%	6.87	0.009^∗^
Female	106	48.0%	12	26.7%		

^∗^ *P* < 0.05; biliary atresia group versus cholestasis group.
