# Supplementary material for: Fat-Soluble Vitamin Deficiency in Pediatric Patients with Biliary Atresia
Source: Gastroenterol Res Pract. 2017 Jun 11;2017:7496860. doi: 10.1155/2017/7496860 (PMC5485346; doi:10.1155/2017/7496860)
Supplement: Supplementary file 1 — Supplementary Table 1: Overall FSV deficiencies in pediatric patients with obstructive jaundice. Supplementary Table 2: Preoperative FSV deficiencies in BA patients. Supplementary Table 3: Comparisons of FSV deficiencies between BA group and cholestatic group. Supplementary Table 4: Preoperative FSV deficiencies in cholestatic patients. Supplementary Table 5: Relationship between preoperative FSV deficiency and sex in BA patients. Supplementary Table 6: FSV deficiencies in different age groups. Supplementary Table 7: Preoperative liver function in BA patients. Supplementary Table 8: Preoperative liver function in cholestatic patients. Supplementary Table 9: Comparison of liver function between the BA and cholestatic groups. Supplementary Table 10: Comparison of liver function among different age groups in BA patients. Supplementary Table 11: Relationship between abnormal preoperative 25-(OH)D level and liver functions in BA patients. Supplementary Table 12: Relationship between preoperative prothrombin time (PT) and liver function in BA patients. Supplementary Table 13: Changes in serum vitamin A level before and after the Kasai procedure in BA patients. Supplementary Table 14: Changes in the serum vitamin D level before and after the Kasai procedure in BA patients. Supplementary Table 15: Changes of serum 25-(OH)D level before and after the Kasai procedure in BA patients. Supplementary Table 16: Changes in serum vitamin E level before and after the Kasai procedure in BA patients. Supplementary Table 17: FSV levels between the low-bilirubin group and the high-bilirubin group 1 month after surgery. Supplementary Table 18: Comparison of vitamin levels between the jaundice-cleared group and the jaundice-non-resolved group 3 months after surgery. Supplementary Table 19: Comparison of vitamin levels between the jaundice-cleared group and the jaundice-non-resolved group 6 months after surgery. [file 7496860.f1.docx]

**Supplementary Table 1: Overall FSV deficiencies in pediatric patients with obstructive jaundice**

| Variables | Interquartile range (IQR) | Deficiency | Percentage of deficiency |
| --- | --- | --- | --- |
| Vitamin A (μmol/L) | 0.85 (0.60 - 1.18) | < 0.52 | 15.2% |
| Vitamin D (nmol/L) | 30.70（24.7 - 38.70） | <25 | 27.0% |
| 25-(OH)D (ng/ml) | 6.96（3.55 - 11.00） | <10 | 87.8% |
| Vitamin E (ng/ml) | 10.97（10.4 - 12.40） | >1.2 | 3.9% |
| International normalized ratio (INR) | 0.96（0.90 - 1.04） | >14.8 | 5.4% |
| Prothrombin time (s) | 12.80（12.5 - 13.70） | <15 | 7.7% |
