# Supplementary material for: Fat-Soluble Vitamin Deficiency in Pediatric Patients with Biliary Atresia
Source: Gastroenterol Res Pract. 2017 Jun 11;2017:7496860. doi: 10.1155/2017/7496860 (PMC5485346; doi:10.1155/2017/7496860)
Supplement: Supplementary file 2 [file 7496860.f2.docx]

**Supplementary Table 2: Preoperative FSV deficiencies in BA patients**

| Variables | Interquartile range (IQR) | Deficiency | Percentage of deficiency |
| --- | --- | --- | --- |
| Vitamin A (μmol/L) | 0.8（0.6 - 1.2） | < 0.52 | 15.6% |
| Vitamin D (nmol/L) | 29.3（23.4 - 37.4） | <25 | 31.3% |
| 25-(OH)D (ng/ml) | 7.7（3.8 - 11.4） | <10 | 88.3% |
| Vitamin E (ng/ml) | 11.0（10.6 - 12.4） | >1.2 | 4.3% |
| International normalized ratio (INR) | 1.0（0.9 - 1.0） | 14.8 | 4.2% |
| Prothrombin time (s) | 12.7（12.1 - 13.6） | <15 | 6.0% |
