# Supplementary material for: Fat-Soluble Vitamin Deficiency in Pediatric Patients with Biliary Atresia
Source: Gastroenterol Res Pract. 2017 Jun 11;2017:7496860. doi: 10.1155/2017/7496860 (PMC5485346; doi:10.1155/2017/7496860)
Supplement: Supplementary file 3 [file 7496860.f3.docx]

**Supplementary Table 3:** Comparisons of FSV deficiencies between BA group and cholestatic group

|  | BA group | Cholestatic group | Chi-Square | *P* value |
| --- | --- | --- | --- | --- |
| Vitamin A | 15.6% | 13.3% | 1.53 | 0.69 |
| Vitamin D | 31.3% | 6.7% | 11.41 | 0.0007* |
| 25-(OH)D | 88.3% | 85.7% | 0.44 | 0.76 |
| Vitamin E | 4.3% | 2.2% | 0.43 | 0.81 |
| International normalized ratio (INR) | 4.2% | 11.1% | 3.50 | 0.073 |
| Prothrombin time (s) | 6.0% | 15.6% | 5.60 | 0.061 |
| Deficiency of any vitamin | 45.9% | 20.0% | 10.27 | 0.0014* |

*P<0.05, BA group *vs.* cholestatic group
