# Supplementary material for: Fat-Soluble Vitamin Deficiency in Pediatric Patients with Biliary Atresia
Source: Gastroenterol Res Pract. 2017 Jun 11;2017:7496860. doi: 10.1155/2017/7496860 (PMC5485346; doi:10.1155/2017/7496860)
Supplement: Supplementary file 4 [file 7496860.f4.docx]

**Supplementary Table 4:** Preoperative FSV deficiencies in cholestatic patients

| Variables | Interquartile range (IQR) | Deficiency | Percentage of deficiency |
| --- | --- | --- | --- |
| Vitamin A (μmol/L) | 0.9（0.6 - 1.2） | < 0.52 | 13.3% |
| Vitamin D (nmol/L) | 37.2（30.4 - 43.4） | <25 | 6.7% |
| 25-(OH)D (ng/ml) | 4.0（3.4 - 8.3） | <10 | 85.7% |
| Vitamin E (ng/ml) | 10.9（10.4 - 12.6） | >1.2 | 2.2% |
| International normalized ratio (INR) | 1.0（0.9 - 1.1） | >14.8 | 11.1% |
| Prothrombin time (s) | 13.1（12.3 - 14.0） | <15 | 15.6% |
