# Supplementary material for: Fat-Soluble Vitamin Deficiency in Pediatric Patients with Biliary Atresia
Source: Gastroenterol Res Pract. 2017 Jun 11;2017:7496860. doi: 10.1155/2017/7496860 (PMC5485346; doi:10.1155/2017/7496860)
Supplement: Supplementary file 5 [file 7496860.f5.docx]

**Supplementary Table 5:** Relationship between preoperative FSV deficiency and sex in BA patients

|  | Males | Females | Chi-Square | *P* value |
| --- | --- | --- | --- | --- |
| Vitamin A | 14.8% | 16.5% | 0.95 | 0.62 |
| Vitamin D | 35.2% | 27.2% | 1.57 | 0.21 |
| 25-(OH)D | 89.6% | 87.0% | 1.47 | 0.52 |
| Vitamin E | 6.5% | 1.9% | 4.25 | 0.12 |
| International normalized ratio (INR) | 4.5% | 3.8% | 0.07 | 1.00 |
| Thrombin time (s) | 6.4% | 5.7% | 1.66 | 0.44 |
| Deficiency of any vitamin | 50% | 41.7% | 1.43 | 0.23 |
