# Supplementary material for: Fat-Soluble Vitamin Deficiency in Pediatric Patients with Biliary Atresia
Source: Gastroenterol Res Pract. 2017 Jun 11;2017:7496860. doi: 10.1155/2017/7496860 (PMC5485346; doi:10.1155/2017/7496860)
Supplement: Supplementary file 6 [file 7496860.f6.docx]

**Supplementary Table 6:** FSV deficiencies in different age groups

|  | 30 - 60-day group  (n=92) | 61 - 90-day group  (n=99) | >90 day group  (n=30) | Chi-Square | *P* value |
| --- | --- | --- | --- | --- | --- |
| Vitamin A | 23.0% | 9.4% | 14.3% | 8.66 | 0.05 |
| Vitamin D | 32.2% | 31.3% | 28.6% | 0.13 | 0.94 |
| 25-(OH)D | 95.0% | 88.9% | 55.6% | 16.30 | 0.0096* |
| Vitamin E | 9.2% | 1.0% | 0.0% | 9.07 | 0.089 |
| International normalized ratio (INR) | 2.2% | 4.1% | 10.7% | 3.84 | 0.15 |
| Thrombin time (s) | 3.3% | 7.2% | 10.7% | 3.72 | 0.42 |
| Deficiency of any vitamin | 52.9% | 40.6% | 42.9% | 2.88 | 0.24 |

　　　*P<0.05, 25-(OH)D deficiency compared among different age groups
