# Supplementary material for: Fat-Soluble Vitamin Deficiency in Pediatric Patients with Biliary Atresia
Source: Gastroenterol Res Pract. 2017 Jun 11;2017:7496860. doi: 10.1155/2017/7496860 (PMC5485346; doi:10.1155/2017/7496860)
Supplement: Supplementary file 7 [file 7496860.f7.docx]

**Supplementary Table 7:** Preoperative liver function in BA patients

| Variables | Normal  value | Mean | Interquartile range (IQR) | Minimum | Maximum |
| --- | --- | --- | --- | --- | --- |
| Total bilirubin (μmol/L) | 5.1-17.1 | 164.9 | 155.7（133.6 - 186.0） | 75.6 | 427.7 |
| Direct bilirubin (μmol/L) | 0-6 | 110.6 | 103.7（91.0 - 124.6） | 49.6 | 276.7 |
| Alkaline phosphatase (IU/L) | 42-383 | 653.3 | 598.0（495.0 - 762.0） | 215.0 | 1723 |
| Glutamine transferase (IU/L) | 7-50 | 809.0 | 661.（297.5 - 1187.5） | 70.0 | 2799 |
| Alanine aminotransferase (IU/L) | 0-40 | 100.7 | 82.0（55.0 - 121.0） | 14.0 | 451.0 |
| Aspartate transaminase (IU/L) | 0-40 | 158.5 | 125.0（95.0 - 178.0） | 29.0 | 719.0 |
| Bile acid (μmol/L) | 0-10 | 143.1 | 134.8（98.4 - 178.9） | 24.1 | 371.9 |
| Albumin (g/L) | 35-55 | 39.1 | 39.1（37.1 - 41.2） | 23.9 | 49.4 |
| Hemoglobin (g/L) | 110-160 | 99.0 | 98.0（91.0 - 104.1） | 63.2 | 146.0 |
| Calcium (mmol/L) | 2.25-2.75 | 2.5 | 2.5（2.4 - 2.6） | 1.8 | 3.2 |
| Phosphorus (mmol/L) | 1.0-1.95 | 2.0 | 2.0（1.9 - 2.2） | 0.8 | 3.3 |
