# Supplementary material for: Fat-Soluble Vitamin Deficiency in Pediatric Patients with Biliary Atresia
Source: Gastroenterol Res Pract. 2017 Jun 11;2017:7496860. doi: 10.1155/2017/7496860 (PMC5485346; doi:10.1155/2017/7496860)
Supplement: Supplementary file 8 [file 7496860.f8.docx]

**Supplementary Table 8:** Preoperative liver function in cholestatic patients

| Variables | Normal  values | Mean | Interquartile range (IQR) | Minimum | Maximum |
| --- | --- | --- | --- | --- | --- |
| Total bilirubin (μmol/L) | 5.1-17.1 | 144.9 | 141.8（117.0 - 177.3） | 10.6 | 272.2 |
| Direct bilirubin (μmol/L) | 0-6 | 98.4 | 96.3（79.1 - 117.7） | 4.6 | 181.4 |
| Alkaline phosphatase (IU/L) | 42-383 | 671.7 | 580.0（474.0 - 771.0） | 236.0 | 1708 |
| Glutamine transferase (IU/L) | 7-50 | 455.2 | 235.0（141.0 - 693.0） | 55.0 | 1890 |
| Alanine aminotransferase (IU/L) | 0-40 | 92.0 | 68.0（45.0 - 86.0） | 23.0 | 539.0 |
| Aspartate transaminase (IU/L) | 0-40 | 139.8 | 110.0（73.0 - 156.0） | 44.0 | 762.0 |
| Bile acid (μmol/L) | 0-10 | 124.7 | 121.8（85.1 - 151.0） | 23.8 | 310.5 |
| Albumin (g/L) | 35-55 | 39.3 | 39.0（36.8 - 41.5） | 33.8 | 48.1 |
| Hemoglobin (g/L) | 110-160 | 103.0 | 102.2（94.0 - 109.0） | 74.0 | 144.0 |
| Calcium (mmol/L) | 2.25-2.75 | 2.5 | 2.5（2.5 - 2.6） | 1.7 | 3.0 |
| Phosphorus (mmol/L) | 1.0-1.95 | 2.0 | 2.1（1.9 - 2.2） | 1.3 | 2.6 |
