# Supplementary material for: Fat-Soluble Vitamin Deficiency in Pediatric Patients with Biliary Atresia
Source: Gastroenterol Res Pract. 2017 Jun 11;2017:7496860. doi: 10.1155/2017/7496860 (PMC5485346; doi:10.1155/2017/7496860)
Supplement: Supplementary file 9 [file 7496860.f9.docx]

**Supplementary Table 9:** Comparison of liver function between the BA and cholestatic groups

|  | BA group (n=221) | Cholestatic group (n=45) |  |  |
| --- | --- | --- | --- | --- |
| Variables | Median (IQR) | Median (IQR) | x^2^ | *P* value |
| Total bilirubin (μmol/L) | 155.7（133.6 - 186.0） | 141.8（117.0 - 177.3） | -2.30 | 0.022 |
| Direct bilirubin (μmol/L) | 103.7（91.0 - 124.6） | 96.3（79.1 - 117.7） | -2.11 | 0.035 |
| Alkaline phosphatase (IU/L) | 598.0（495.0 - 762.0） | 580.0（474.0 - 771.0） | -0.22 | 0.83 |
| Glutamine transferase (IU/L) | 661.0（297.5 - 1187.5） | 235.0（141.0 - 693.0） | -4.39 | <0.0001 |
| Alanine aminotransferase (IU/L) | 82.0（55.0 - 121.0） | 68.0（45.0 - 86.0） | -1.65 | 0.10 |
| Aspartate transaminase (IU/L) | 125.0（95.0 - 178.0） | 110.0（73.0 - 156.0） | -2.06 | 0.040 |
| Bile acid (μmol/L) | 134.8（98.4 - 178.9） | 121.8（85.1 - 151.0） | -1.90 | 0.058 |
| Albumin (g/L) | 39.1（37.1 - 41.2） | 39.0（36.8 - 41.5） | 0.03 | 0.97 |
| Hemoglobin (g/L) | 98.0（91.0 - 104.1） | 102.2（94.0 - 109.0） | 2.05 | 0.041 |
| Calcium (mmol/L) | 2.5（2.4 - 2.6） | 2.5（2.5 - 2.6） | 0.86 | 0.39 |
| Phosphorus (mmol/L) | 2.0（1.9 - 2.2） | 2.1（1.9 - 2.2） | 0.81 | 0.42 |
