# Supplementary material for: Fat-Soluble Vitamin Deficiency in Pediatric Patients with Biliary Atresia
Source: Gastroenterol Res Pract. 2017 Jun 11;2017:7496860. doi: 10.1155/2017/7496860 (PMC5485346; doi:10.1155/2017/7496860)
Supplement: Supplementary file 10 [file 7496860.f10.docx]

**Supplementary Table 10:** Comparison of liver function among different age groups in BA patients

|  | 30 - 60-day group (n=92) | 61 - 90-day group (n=99) | >90-day group (n=32) |  |  |
| --- | --- | --- | --- | --- | --- |
|  | Median (IQR) | Median (IQR) | Median (IQR) | x2 | *P* value |
| Total bilirubin (μmol/L) | 155.1（138.45 - 191.5） | 154.2（132.6 - 181.7） | 157.9（123 - 189.1） | 0.16 | 0.93 |
| Direct bilirubin (μmol/L) | 102.1（91.1 - 124.25） | 104.3（90.6 - 125.1） | 105.65（92 - 134.5） | 0.20 | 0.91 |
| Alkaline phosphatase (IU/L) | 569.5（484 - 647） | 619（513 - 832） | 726.5（534 - 984） | 10.42 | 0.006^*^ |
| Glutamine transferase (IU/L) | 497（245 - 934.5） | 751.5（346 - 1230） | 1156.5（522 - 1452） | 14.91 | 0.0006^*^ |
| Alanine aminotransferase (IU/L) | 66.5（52.5 - 102） | 88（55 - 134） | 113（78 - 159） | 10.61 | 0.005^*^ |
| Aspartate transaminase (IU/L) | 114.5（80.5 - 164） | 132（98 - 182） | 164.5（114 - 269） | 14.57 | 0.0007^*^ |
| Bile acid (μmol/L) | 124.9（98 - 160） | 139.3（98.2 - 179.7） | 177.55（130.1 - 240.9） | 11.68 | 0.0029^*^ |
| Albumin (g/L) | 38.7（36.95 - 40.85） | 39.15（37.6 - 41.5） | 40.15（37.5 - 43） | 3.12 | 0.21 |
| Hemoglobin (g/L) | 98（90.6 - 105.3） | 97.5（91 - 104） | 98.5（90 - 104） | 0.05 | 0.98 |
| Calcium (mmol/L) | 2.53（2.44 - 2.62） | 2.49（2.39 - 2.6） | 2.47（2.36 - 2.6） | 4.38 | 0.11 |
| Phosphorus (mmol/L) | 2.09（1.95 - 2.21） | 1.98（1.81 - 2.11） | 1.91（1.54 - 2.08） | 15.13 | 0.0005^*^ |
