# Supplementary material for: Fat-Soluble Vitamin Deficiency in Pediatric Patients with Biliary Atresia
Source: Gastroenterol Res Pract. 2017 Jun 11;2017:7496860. doi: 10.1155/2017/7496860 (PMC5485346; doi:10.1155/2017/7496860)
Supplement: Supplementary file 11 [file 7496860.f11.docx]

**Supplementary Table11:** Relationship between abnormal preoperative 25-(OH)D level and liver functions in BA patients

| Liver functions | Correlation coefficients with 25-(OH)D | *P* value |
| --- | --- | --- |
| Total bilirubin | 0.163 | 0.117 |
| Direct bilirubin (μmol/L) | 0.163 | 0.115 |
| Alkaline phosphatase | 0.060 | 0.568 |
| γ-glutamyl transferase (IU/L) | 0.223 | 0.031* |
| Alanine aminotransferase | 0.036 | 0.728 |
| Aspartate aminotransferase | 0.193 | 0.062 |
| Bile acid | 0.210 | 0.042* |
| Albumin | 0.147 | 0.160 |
| Hemoglobin | 0.142 | 0.172 |
| Calcium | 0.281 | 0.006* |
| Phosphorus | 0.063 | 0.550 |

*P<0.05, when analyzing the correlation between liver function and 25-(OH)D
