# Supplementary material for: Fat-Soluble Vitamin Deficiency in Pediatric Patients with Biliary Atresia
Source: Gastroenterol Res Pract. 2017 Jun 11;2017:7496860. doi: 10.1155/2017/7496860 (PMC5485346; doi:10.1155/2017/7496860)
Supplement: Supplementary file 12 [file 7496860.f12.docx]

**Supplementary Table 12:** Relationship between preoperative prothrombin time (PT) and liver function in BA patients

| Liver functions | Correlation coefficients with PT (r value) | *P* value |
| --- | --- | --- |
| Total bilirubin | -0.016 | 0.814 |
| Direct bilirubin (μmol/L) | -0.033 | 0.628 |
| Alkaline phosphatase (ALP) | 0.310 | <.0001* |
| γ-glutamyl transferase (IU/L) | -0.291 | <.0001* |
| Alanine aminotransferase | 0.208 | 0.002* |
| Aspartate aminotransferase | 0.232 | 0.001* |
| Bile acid | 0.009 | 0.8953 |
| Albumin | -0.378 | <0.0001* |
| Hemoglobin | 0.113 | 0.100 |
| Calcium | -0.312 | <0.0001* |
| Phosphorus | -0.016 | 0.814 |

*P<0.05, when analyzing the correlation between liver function and PT
