# Supplementary material for: Fat-Soluble Vitamin Deficiency in Pediatric Patients with Biliary Atresia
Source: Gastroenterol Res Pract. 2017 Jun 11;2017:7496860. doi: 10.1155/2017/7496860 (PMC5485346; doi:10.1155/2017/7496860)
Supplement: Supplementary file 13 [file 7496860.f13.docx]

**Supplementary Table 13:** Changes in serum vitamin A level before and after the Kasai procedure in BA patients

|  |  | Mean | Median (IQR) |
| --- | --- | --- | --- |
| Vitamin A  (μmol/L) | Before surgery | 0.97 | 0.93（0.74 - 1.19） |
|  | 2 weeks after surgery | 0.87 | 0.78（0.60 - 1.08） |
|  | 1 month after surgery | 0.81 | 0.66（0.57 - 0.98） |
|  | 3 months after surgery | 0.76 | 0.64（0.57 - 0.85） |
|  | 6 months after surgery | 0.75 | 0.64（0.58 - 0.87） |
