# Supplementary material for: Fat-Soluble Vitamin Deficiency in Pediatric Patients with Biliary Atresia
Source: Gastroenterol Res Pract. 2017 Jun 11;2017:7496860. doi: 10.1155/2017/7496860 (PMC5485346; doi:10.1155/2017/7496860)
Supplement: Supplementary file 14 [file 7496860.f14.docx]

**Supplementary Table 14:** Changes in the serum vitamin D level before and after the Kasai procedure in BA patients

|  |  | Mean | Median (IQR) |
| --- | --- | --- | --- |
| Vitamin D  (nmol/L) | Before surgery | 36.14 | 34.25（26.86 - 41.66） |
|  | 2 weeks after surgery | 48.93 | 40.08（35.48 - 63.43） |
|  | 1 months after surgery | 35.26 | 32.28（28.9 - 40.84） |
|  | 3 months after surgery | 35.16 | 34.22（30.19 - 40.27） |
|  | 6 months after surgery | 33.08 | 31.2（28.51 - 38.25） |
