# Supplementary material for: Fat-Soluble Vitamin Deficiency in Pediatric Patients with Biliary Atresia
Source: Gastroenterol Res Pract. 2017 Jun 11;2017:7496860. doi: 10.1155/2017/7496860 (PMC5485346; doi:10.1155/2017/7496860)
Supplement: Supplementary file 15 [file 7496860.f15.docx]

**Supplementary Table 15:** Changes of serum 25-(OH)D level before and after the Kasai procedure in BA patients

|  |  | Mean | Median (IQR) |
| --- | --- | --- | --- |
| 25-(OH)D  (ng/ml) | Before surgery | 8.75 | 7.66（3.75 - 11.35） |
|  | 2 weeks after surgery | 5.37 | 4.04（3 - 6.52） |
|  | 1 months after surgery | 7.07 | 5.13（3 - 9.37） |
|  | 3 months after surgery | 17.6 | 15.55（7.53 - 25.3） |
|  | 6 months after surgery | 19.16 | 13.91*（9.69 - 28.78） |
