# Supplementary material for: Fat-Soluble Vitamin Deficiency in Pediatric Patients with Biliary Atresia
Source: Gastroenterol Res Pract. 2017 Jun 11;2017:7496860. doi: 10.1155/2017/7496860 (PMC5485346; doi:10.1155/2017/7496860)
Supplement: Supplementary file 16 [file 7496860.f16.docx]

**Supplementary Table 16:** Changes in serum vitamin E level before and after the Kasai procedure in BA patients

|  |  | Mean | Median (IQR) |
| --- | --- | --- | --- |
| Vitamin E  (ng/ml) | Before surgery | 11.41 | 10.98（10.55 - 11.81） |
|  | 2 weeks after surgery | 11.27 | 10.72（10.17 - 11.93） |
|  | 1 months after surgery | 10.81 | 10.57（10.3 - 11.04） |
|  | 3 months after surgery | 10.55 | 10.5（10.21 - 10.78） |
|  | 6 months after surgery | 11.3 | 11.4（10.7 - 11.84） |
