# Supplementary material for: Fat-Soluble Vitamin Deficiency in Pediatric Patients with Biliary Atresia
Source: Gastroenterol Res Pract. 2017 Jun 11;2017:7496860. doi: 10.1155/2017/7496860 (PMC5485346; doi:10.1155/2017/7496860)
Supplement: Supplementary file 17 [file 7496860.f17.docx]

**Supplementary Table 17:** FSV levels between the low-bilirubin group and the high-bilirubin group 1 month after surgery

|  |  | Low-bilirubin group | High-bilirubin group |  |  |
| --- | --- | --- | --- | --- | --- |
| Variables | Time | Interquartile range (IQR) | Interquartile range (IQR) | Z | P |
|  | Before surgery | 0.90（0.68 - 1.12） | 0.95（0.73 - 1.25） | -0.59 | 0.56 |
| Vitamin A | 1 months after surgery | 0.56（0.54 - 0.64） | 0.83（0.61 - 1.15） | -3.34 | 0.0008* |
|  | Difference | -0.17（-0.52 - -0.06） | -0.17（-0.43 - 0.20） | -1.37 | 0.17 |
|  | Before surgery | 11.03（10.59 - 11.21） | 10.80（10.41 - 11.51） | 0.59 | 0.56 |
| Vitamin E | 1 months after surgery | 10.37（10.26 - 10.77） | 10.69（10.33 - 11.40） | -1.71 | 0.088 |
|  | Difference | -0.54（-1.01 - 0.17） | -0.08（-0.86 - 0.46） | -1.55 | 0.12 |
|  | Before surgery | 37.05（27.52 - 40.43） | 33.14（26.05 - 42.14） | 0.81 | 0.42 |
| Vitamin D | 1 months after surgery | 31.82（29.52 - 35.64） | 32.79（29.27 - 40.09） | -0.29 | 0.77 |
|  | Difference | -4.26（-8.08 - 4.30） | 2.10（-5.78 - 9.05） | -0.89 | 0.37 |
|  | Before surgery | 5.95（3.72 - 8.45） | 8.27（3.75 - 11.34） | -0.68 | 0.50 |
| 25-(OH)D | 1 months after surgery | 7.44（3.00 - 11.03） | 5.08（3.00 - 7.81） | 1.16 | 0.24 |
|  | Difference | 0.05（-0.80 - 7.17） | -1.99（-3.83 - -0.15） | 2.63 | 0.0086* |

Note: The measurement units for vitamins A, E, and D and 25-(OH)D were μmol/L, ng/ml, nmol/L, and ng/ml, respectively.

*P<0.05, low-bilirubin group *vs.* high-bilirubin group
