# Supplementary material for: Fat-Soluble Vitamin Deficiency in Pediatric Patients with Biliary Atresia
Source: Gastroenterol Res Pract. 2017 Jun 11;2017:7496860. doi: 10.1155/2017/7496860 (PMC5485346; doi:10.1155/2017/7496860)
Supplement: Supplementary file 18 [file 7496860.f18.docx]

**Supplementary Table 18:** Comparison of vitamin levels between the jaundice-cleared group and the jaundice-non-resolved group 3 months after surgery

|  |  | jaundice-cleared group | jaundice-non-resolved group |  |  |
| --- | --- | --- | --- | --- | --- |
| Variables | Time | Interquartile range (IQR) | Interquartile range (IQR) | Z | P |
|  | Before surgery | 0.93（0.68 - 1.12） | 0.81（0.72 - 0.97） | -0.69 | 0.49 |
| Vitamin A | 3 months after surgery | 0.60（0.54 - 0.67） | 0.93（0.64 - 1.22） | 3.37 | 0.0007* |
|  | Difference | -0.34（-0.53 - -0.07） | 0.09（-0.17 - 0.48） | 2.84 | 0.0045* |
|  | Before surgery | 10.81（10.39 - 11.12） | 10.89（10.57 - 11.57） | 1.02 | 0.31 |
| Vitamin E | 3 months after surgery | 10.47（10.17 - ） | 10.55（10.21 - 10.94） | 0.57 | 0.57 |
|  | Difference | -0.33（10.76 - -0.96） | -0.47（-1.18 - 0.01） | -0.49 | 0.62 |
|  | Before surgery | 35.29（41.23 - ） | 37.73（28.57 - 45.24） | 0.83 | 0.41 |
| Vitamin D | 3 months after surgery | 36.69（30.80 - 40.94） | 33.37（31.38 - 35.63） | -1.23 | 0.22 |
|  | Difference | 2.41（-6.55 - 7.54） | -7.43（-11.14 - 1.77） | -1.65 | 0.10 |
|  | Before surgery | 7.74（3.76 - 11.34） | 10.98（7.92 - 15.58） | 1.98 | 0.047* |
| 25-(OH)D | 3 months after surgery | 24.18（13.75 - 29.97） | 8.25（5.13 - 15.55） | -3.16 | 0.0016* |
|  | Difference | 17.52（4.99 - 21.49） | -0.83（-5.96 - 1.44） | -3.63 | 0.0003* |

Note: The measurement units for vitamins A, E, and D and 25-(OH)D were μmol/L, ng/ml, nmol/L, and ng/ml, respectively. *P<0.05, jaundice-cleared group *vs.* jaundice-non-resolved group
