# Supplementary material for: Fat-Soluble Vitamin Deficiency in Pediatric Patients with Biliary Atresia
Source: Gastroenterol Res Pract. 2017 Jun 11;2017:7496860. doi: 10.1155/2017/7496860 (PMC5485346; doi:10.1155/2017/7496860)
Supplement: Supplementary file 19 [file 7496860.f19.docx]

**Supplementary Table 19:** Comparison of vitamin levels between the jaundice-cleared group and the jaundice-non-resolved group 6 months after surgery

|  |  | jaundice-cleared group | jaundice-non-resolved group |  |  |
| --- | --- | --- | --- | --- | --- |
| Variables | Time | Interquartile range (IQR) | Interquartile range (IQR) | Z | P |
|  | Before surgery | 0.98（0.74 - 1.12） | 1.00（0.88 - 1.15） | 0.28 | 0.78 |
| Vitamin A | 6 months after surgery | 0.61（0.55 - 0.82） | 0.87（0.71 - 0.98） | 2.39 | 0.017* |
|  | Difference | -0.23（-0.57 - -0.04） | -0.12（-0.36 - 0.17） | 1.25 | 0.21 |
|  | Before surgery | 10.60（10.26 - 11.21） | 10.46（10.42 - 10.83） | -0.17 | 0.87 |
| Vitamin E | 6 months after surgery | 11.43（10.68 - 11.83） | 11.30（10.73 - 11.94） | -0.08 | 0.93 |
|  | Difference | 0.67（0.26 - 1.44） | 0.67（0.01 - 1.25） | -0.03 | 0.98 |
|  | Before surgery | 31.39（26.89 - 39.84） | 29.78（25.22 - 42.23） | -0.25 | 0.80 |
| Vitamin D | 6 months after surgery | 34.13（30.33 - 41.40） | 27.56（25.73 - 29.20） | -3.25 | 0.0012* |
|  | Difference | 3.35（-8.11 - 13.86） | -1.95（-13.74 - 1.56） | -1.58 | 0.11 |
|  | Before surgery | 5.95（3.00 - 11.79） | 8.95（5.51 - 13.81） | 0.92 | 0.36 |
| 25-(OH)D3 | 6 months after surgery | 21.91（14.06 - 34.16） | 8.76（6.71 - 11.43） | -3.42 | 0.0006* |
|  | Difference | 13.19（9.32 - 24.69） | -1.92（-4.89 - 3.11） | -3.31 | 0.0009* |

Note: The measurement units for vitamins A, E, and D and 25-(OH)D were μmol/L, ng/ml, nmol/L, and ng/ml, respectively.

*P<0.05, jaundice-cleared group *vs.* jaundice-non-resolved group
